# Prevalence and risk factors of human trichostrongylosis in Satun, southern Thailand

**DOI:** 10.1051/parasite/2026027

**Published:** 2026-05-25

**Authors:** Teera Kusolsuk, Thongroo Kophachon, Surapol Sa-Nguankiat, Nirundorn Homsuwan, Wichuda Lappuechudom, Supatta Srithongtae, Oranard Wattanawong, Yukifumi Nawa, Sivapong Sungpradit

**Affiliations:** 1 Department of Helminthology, Faculty of Tropical Medicine, Mahidol University Bangkok Thailand; 2 Division of Communicable Diseases, Department of Disease Control, Ministry of Public Health Nonthaburi Thailand; 3 Office of Senior Advisory Committee, Department of Health, Ministry of Public Health Nonthaburi Thailand; 4 Office of Diseases Prevention and Control 4 Saraburi, Ministry of Public Health Saraburi Thailand; 5 Tropical Diseases Research Centre, Faculty of Medicine, Khon Kaen University Khon Kaen Thailand; 6 Department of Pre-Clinic and Applied Animal Science, Faculty of Veterinary Science, Mahidol University Nakhon Pathom Thailand

**Keywords:** PCR-RFLP, Risk factor, Ruminant, *Trichostrongylus*

## Abstract

*Trichostrongylus* spp., zoonotic soil-transmitted nematodes, affect both livestock and humans globally. In Thailand, human trichostrongylosis has been reported, but no systematic study examining livestock and humans in the same locality has been conducted. This study aimed to address this gap in Satun Province, southern Thailand by characterizing the disease’s epidemiologic status among livestock, farmers, and the environment using both microscopic and molecular techniques. Human risk factors were assessed via questionnaires. Stool samples were collected from livestock farmers, their families, and herbivores in Nongkhai, Ratchaburi, and Satun Provinces. Vegetable samples were also obtained from households and local markets. Human and livestock feces were examined using the modified Kato–Katz method and a Mini Parasep^®^ solvent-free fecal parasite concentrator. Total DNA was then extracted from fecal and vegetable sediments, and a 211 bp fragment of the ribosomal internal transcribed spacer 2 (*ITS2*) gene was amplified using a polymerase chain reaction (PCR). PCR products were digested with *Hinf*I and analyzed via electrophoresis to identify *Trichostrongylus* species. In Satun, *Trichostrongylus colubriformis* monoinfection was found in 12 of 221 (5.4%) residents, while co-infection with *T. colubriformis* and *T. axei* occurred in 3 (1.4%). Herbivore feces and vegetable sediments from Satun also tested positive. No human cases were found in Nongkhai or Ratchaburi. Risk factors included education level, occupation, and outdoor toilet use. Most infected individuals in Satun were asymptomatic. Despite this, public health interventions should be implemented, targeting the human–livestock–environment interface to control the disease effectively.

## Abstract (fr)

*Trichostrongylus* spp., des nématode zoonotique transmis par le sol, affectent le bétail et les humains à l'échelle mondiale. En Thaïlande, des cas de trichostrongylose humaine ont été rapportés, mais aucune étude systématique n'a été menée sur le bétail et les humains dans une même localité. Cette étude visait à combler cette lacune dans la province de Satun, dans le sud de la Thaïlande, en caractérisant le statut épidémiologique de la maladie chez le bétail, les agriculteurs et dans l'environnement à l'aide de techniques microscopiques et moléculaires. Les facteurs de risque humains ont été évalués par questionnaire. Des échantillons de selles ont été prélevés auprès d'éleveurs, de leurs familles et d'animaux herbivores dans les provinces de Nongkhai, Ratchaburi et Satun. Des échantillons de légumes ont également été obtenus auprès des ménages et sur les marchés locaux. Les selles humaines et animales ont été examinées à l'aide de la méthode de Kato-Katz modifiée et du concentrateur de parasites fécaux sans solvant Mini Parasep^®^. L'ADN total a ensuite été extrait des sédiments fécaux et végétaux, et un fragment de 211 pb du gène ITS2 (espaceur transcrit interne 2) de l'ARN ribosomique a été amplifié par PCR. Les produits de PCR ont été digérés par l'enzyme HinfI et analysés par électrophorèse pour identifier l'espèce de *Trichostrongylus*. À Satun, une mono-infection à *Trichostrongylus colubriformis* a été observée chez 12 des 221 résidents (5,4 %), tandis qu'une co-infection par *T. colubriformis* et *T. axei* a été constatée chez 3 d'entre eux (1 %). Les excréments d'herbivores et les sédiments végétaux de Satun se sont également révélés positifs. Aucun cas humain n'a été recensé à Nongkhai ni à Ratchaburi. Les facteurs de risque comprenaient le niveau d'instruction, la profession et l'utilisation de toilettes extérieures. La plupart des personnes infectées à Satun étaient asymptomatiques. Malgré cela, des interventions de santé publique, ciblant l'interface homme-bétail-environnement, devraient être mises en œuvre afin de contrôler efficacement la maladie.

## Introduction

Human trichostrongylosis, a soil-transmitted helminthic infection caused by nematodes of the Trichostrongylidae family, remains a significant yet underreported zoonotic disease [52]. Its global distribution has been documented in countries such as Australia, Brazil, Chile, China, France, Ghana, India, Iran, Israel, Italy, Lao PDR, Rwanda, Tunisia [2, 8, 37, 48], and Thailand [27, 39, 41, 42]. Human transmission primarily occurs through ingestion of raw or improperly washed vegetables or water contaminated with feces from small ruminants like goats and sheep, which contain infective third-stage larvae (L3) [6, 24, 56]. Rarely, L3 larvae may also penetrate the skin and migrate to the intestines, where they develop into adult worms [20]. Zoonotic species such as *Trichostrongylus colubriformis*, *T. axei*, and *T. vitrinus* are the primary agents of human infection, with adult worms residing in the small intestine. Clinical symptoms include abdominal pain, diarrhea, eosinophilia, and hypochromic microcytic anemia, although most cases remain asymptomatic [11, 15, 19, 56, 58].

While trichostrongylosis is well-documented in livestock such as goats and sheep [8], the overlap between human and animal infections underscores the parasite’s zoonotic potential. Deworming programs and improved farm management practices have been implemented to control its spread among livestock [29]. However, these efforts are increasing challenged by the rise of anthelmintic resistance. Resistance has been reported to benzimidazoles (e.g., albendazole, fenbendazole), macrocyclic lactones (e.g., ivermectin, moxidectin), and imidazothiazoles (e.g., levamisole), with global evidence of emerging multidrug resistance [16, 34]. Benzimidazole drugs such as albendazole and mebendazole remain the standard treatment for human trichostrongylosis [5]. However, the increasing prevalence of anthelmintic resistance may result in persistent infections despite treatment, potentially leading to an underestimation of the true disease burden [50].

This study aimed to determine the prevalence and associated risk factors of trichostrongylosis among livestock farmers, their families, livestock, and fresh vegetables in Satun Province, southern Thailand, as well as in two additional provinces located in northeastern and central Thailand. These regions were selected due to their high density of goat farms, a recognized risk factor for zoonotic trichostrongylosis in humans. By employing both microscopic and molecular techniques, the study sought to identify *Trichostrongylus* species and evaluate their distribution. The findings are expected to provide valuable insights for public health interventions and help raise awareness about the risks associated with soil-transmitted helminths in the region.

## Materials and methods

### Ethics approval

This study was approved by the human ethics committee of the Faculty of Tropical Medicine, Mahidol University (certificate of ethics approval: MUTM-2020-064-01). All participants (>15 years of age) signed the inform consent before providing the stool samples and questionnaires. The study on livestock was approved by the animal ethics committee (COA No. MUVS-2020-07-25) and biosafety committee (IBC/MUVS-B-010/2563) of the Faculty of Veterinary Science, Mahidol University.

### Study area

**Figure 1:**
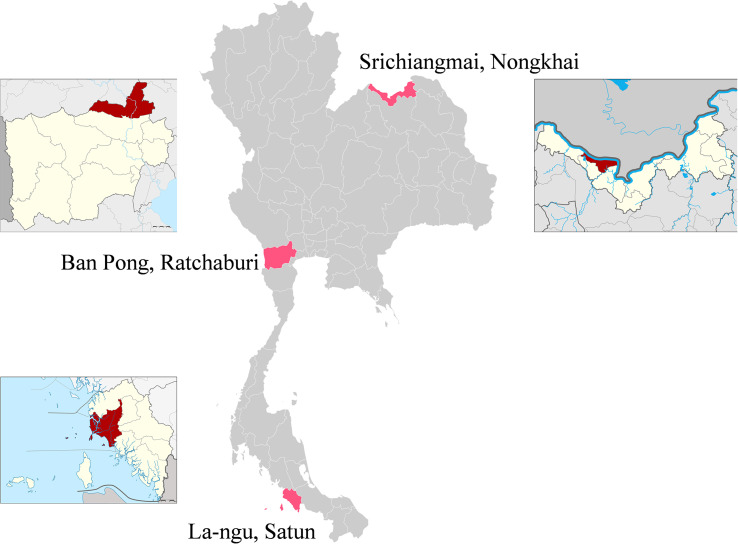
Map of Thailand showing the three sample collection sites used in this study for the 445 human and 342 livestock fecal samples.

Samples were collected between September 2020 and April 2021. The selected cross-sectional study sites included: Sri-Chiangmai District, Nongkhai Province (northeastern Thailand; 17°57′23.8″N, 102°35′26.7″E); Nong-Kop Subdistrict, Ban Pong District, Ratchaburi Province (central Thailand; 13°49′04.6″N, 99°56′11.6″E); and La-ngu Subdistrict, La-ngu District, Satun Province (southern Thailand; 6°55′53.1″N, 99°47′11.5″E, and 6°51′37.6″N, 99°47′34.1″E) (Fig. 1).

### Stool examination and DNA extraction

Approximately 5–10 g of each human stool specimen were collected in labeled plastic bags and handed over to healthcare volunteers, then stored at 4 °C until further processing. Fresh specimens were examined for helminth infections using the modified Kato–Katz thick smear method [38]. Samples were filtered through a 0.5–1 mm mesh to remove debris and dietary fibers. About 100–150 mg of filtered stool was placed on a glass slide, covered with cellophane soaked in glycerin-malachite green, pressed to flatten the sample, and allowed to dry for 15–30 min before microscopic observation.

Livestock stool samples were examined using a Mini Parasep^®^ solvent-free (SF) kit, following the manufacturer’s instructions. Briefly, fecal samples were scooped and mixed with 3.3 mL of 10% buffered formalin and Triton X, then vortexed thoroughly. The Mini Parasep^®^ tubes were centrifuged at 200 × *g* for 2 min. After discarding the supernatant, a drop of the sediment was mixed with normal saline on a glass slide and examined microscopically.

DNA extraction from both 445 human and 342 livestock stool samples was performed using a QIAamp^®^ Fast DNA Stool Mini Kit (QIAGEN, Hilden, Germany), with slight modifications to the manufacturer’s protocol. Approximately 500 mg of stool were homogenized using 0.5 mm glass beads (Omni International Inc., Kennesaw, GA, USA). Homogenization was performed with a Bead Ruptor Elite homogenizer at 3 m/s for two 30-second cycles, separated by a 30-second pause. Eluted DNA was stored at −20 °C for further molecular analysis.

### Vegetable examination and DNA extraction

Approximately 200 g of each vegetable sample was minced using a sterile knife and cutting board, then soaked in a beaker containing 500 mL of 0.85% NaCl for 20 min. This was followed by agitation on a shaker for 5 min to ensure thorough washing. After washing, the vegetable pieces were removed, and the wash solution was allowed to stand undisturbed for 1 h to facilitate sedimentation of parasitic stages. The upper layer of saline was carefully decanted without disturbing the sediment. The remaining sediment was centrifuged at 2,000× *g* for 5 min. The final sediment was then examined microscopically for parasite eggs and larvae. DNA extraction from vegetable sediments (74 samples) was performed using a DNeasy^®^ PowerSoil^®^ Pro Kit (QIAGEN), with slight modifications to the manufacturer’s protocol.

### PCR-restriction fragment length polymorphism (PCR-RFLP) of *Trichostrongylus* species

DNA extracted from stool and vegetable sediment samples was used as a template to amplify a 211-base pair (bp) fragment of the *Trichostrongylus* internal transcribed spacer 2 (ITS2*)* region of ribosomal DNA. *Trichostrongylus* genus-specific primers used were (Tri-F 5′–AATGAATTTCTACAGTGTGG-3′ and Tri-R 5′-CATACATGTCCCTGTTTAAATC-3′), as described by Mizani *et al.* [35] and synthesized by Macrogen Laboratory, (Seoul, South Korea).

The PCR reaction was prepared in a final volume of 50 μL, containing 25 μL of TopTaq Master Mix (QIAGEN), 1.0 μL each of forward and reverse primers (10 μM), 5.0 μL of CoralLoad Concentrate (QIAGEN), 5.0 μL of DNA template, and molecular biology-grade water (QIAGEN) to reach the final volume. Reactions were performed in PCR tubes (Corning, Axygen Scientific, Union City, CA, USA), using a T100^TM^ Thermal Cycler (Bio-Rad, Hercules, CA, USA). Thermocycling conditions were as follows: initial denaturation at 95 °C for 5 min, followed by 35 cycles of denaturation at 95 °C for 45 s, annealing at 55 °C for 45 s, and 60 s extension at 72 °C for 60 s, with a final extension at 72 °C for 7 min.

PCR products of the *Trichostrongylus* ITS2 region were subjected to restriction enzyme digestion using *Hidf*I which recognizes the sequence of G/ANTC (New England Biolabs, Ipswich, MA, USA). The reaction was carried out in a total volume of 20 μL, containing 15 μL of PCR product, 2 μL of 10× rCutSmart buffer, 0.5 μL (5 units) of *Hidf*I enzyme, and 2.5 μL of DNase-free water, following the protocol described by Mizani *et al.* [35]. The reaction mixtures were incubated at 37 °C in a water bath for 1 h. Digested products were separated on a 3.0% agarose gel in 1× TBE buffer at 100 V for 45 min, stained with GelRed^TM^ (Biotium, Hayward, CA, USA), and visualized under UV light using a transilluminator (Azure Biosystems, Dublin, CA, USA). Selected PCR products of the ITS2 region were then sequenced using both Tri-F and Tri-R primers to confirm species of *Trichostrongylus*. The resulting sequences were compared with reference sequences in the GenBank database using the Basic Local Alignment Search Tool (BLAST) (accessed on January 9, 2025) [3].

### PCR of hookworm DNA

To amplify a 597 bp fragment of the ITS1–5.8S rRNA–ITS2 region of hookworm species DNA (*Necator americanus* and *Ancylostoma* spp.), the primer set UGHWF (5′-GTTGGGAGTATCRCCMMCCK-3′) and NC1R (5′-AACAACCCTGAACCAGACGT-3′) was used, as described by George *et al.* [17]. To verify the hookworm species, selected PCR products encompassing the ITS1–5.8S rRNA–ITS2 region were sequenced using the UGHWF and NC1R primers. The obtained sequences were subsequently aligned and compared against reference data in the GenBank database via BLAST (accessed on January 9, 2025) [3].

### Nested PCR and allele-specific polymerase chain reaction (AS-PCR)

To investigate the benzimidazole resistance phenotype, F200Y polymorphism in the isotype 1 β-tubulin gene was analyzed. Third-stage *Trichostrongylus* larvae from five positive human trichostrongylosis cases were enriched using the Harada–Mori fecal culture method [40]. DNA was extracted from individual larvae using an alkaline lysis method. Under a stereo microscope, a single larva was isolated using the tip of a 26-gauge needle and transferred to a thin-walled PCR tube containing 5 μL of lysis buffer (0.05% SDS and 0.025 N NaOH). The samples were incubated at 95 °C for 15 min in a block heater, centrifuged briefly, then diluted to 100 μL with nuclease-free water, and stored at –20 °C until use [54]. Nested PCR and AS-PCR was performed as described by Silvestre and Humbert [49], and Humbert and Elard [26]. Bands corresponding to the resistant allele (∼250 bp), susceptible allele (∼550 bp), and internal control (∼750 bp) were used to classify genotypes as homozygous resistant (RR), homozygous susceptible (SS), or heterozygous (SR).

### Questionnaire survey

Livestock farmers and their families were invited to participate in this study after providing informed consent. Participants completed questionnaires that captured personal information, routine behaviors, and potential risk factors for helminth infection. The questionnaire consisted of 21 main questions (Supplementary Table 1).

### Statistical analysis

The prevalence of infection was calculated using the formulation of prevalence (%) = number of positives / total number of samples × 100 and the confidence interval of proportion was calculated by sample size calculators for designing clinical research (https://sample-size.net/confidence-interval-proportion/) [30]. Associations between helminth infection status (positive/negative) and demographic or behavioral factors such as gender, religion, education, occupation, and other risk factors were analyzed using the Statistical Package for the Social Science (SPSS) version 22.0 for Windows (SPSS Inc., Chicago, IL, USA). Qualitative data were assessed using Pearson’s Chi-square test. A 95% confidence level was applied to all analyses, and a *p*-value of <0.05 was considered statistically significant.

## Results

### Study sites climate data

Climate data were obtained from www.accuweather.com, and population statistics were sourced from the National Statistical Office of Thailand ([www.nso.go.th] (http://www.nso.go.th)). Sri-Chiangmai District has a tropical climate, with temperatures ranging from 24–33 °C in September 2020 and average rainfall of 184.4 mm. The district had approximately 5,861 households, averaging 4.3 people per household. Ban Pong District has tropical, humid climate, with October 2020 temperatures ranging from 25–31 °C and average rainfall of 175.5 mm. It had around 2,272 households, with an average of 3.4 people per household. La-ngu District also has a tropical humid climate. In October 2020 and April 2021, temperatures ranged from 25.5–30 °C and 25–33 °C, respectively, with average rainfall of 237.2 mm and 115.8 mm. The area had approximately 5,266 households, with an average of four people per household.

### Sample collection

The collected samples included human and livestock stool, vegetables, and questionnaires. A total of 445 human stool samples were obtained from livestock farmers: 121 from Nongkhai, 103 from Ratchaburi, and 221 from Satun Provinces. In total, 342 livestock fecal samples, mainly from goats and cattle, were collected directly from the ground immediately after defecation at farmers’ household: 59 from Nongkhai, 114 from Ratchaburi, and 169 from Satun. Additionally, 74 fresh above-ground vegetable samples were collected: 23 from Nongkhai, 11 from Ratchaburi, and 40 from Satun, from both planting areas and local markets. All morning stool samples were placed in labeled plastic containers, stored in ice cooled boxes, and transported to local laboratories for microscopic examination. Aliquots of each sample were preserved in 90% ethanol for subsequent DNA extraction. Vegetable samples were similarly kept on ice and shipped to the Department of Helminthology, Faculty of Tropical Medicine, Mahidol University, Bangkok.

### Microscopic examination

The comprehensive microscopic findings, categorized by both overall infection rates and specific geographic locations, are detailed in Tables 1 and 2. Additionally, microscopic screening of vegetable sediment revealed the presence of unidentified larvae, with the specific distribution across sampling sites documented in Table 3.

**Table 1:** Microscopic examination of human stool samples using the modified Kato–Katz technique.

Type of ova	Sample size	Nongkhai			Ratchaburi			Satun			Infected cases	Infection rate (%)	95% CI (%)
		Male (%)	Female (%)	Total (%)	Male (%)	Female (%)	Total (%)	Male (%)	Female (%)	Total (%)			
*Opisthorchis viverrini*-like	445	2/76 (2.6)	0/45 (0)	2/121 (1.6)	1/69 (1.4)	0/34 (0)	1/103 (1.0)	0/109 (0)	0/112 (0)	0/221 (0)	3	0.7	0.1–2.0
Minute intestinal fluke	445	3/76 (3.9)	0/45 (0)	3/121 (2.5)	0/69 (0)	1/34 (2.9)	1/103 (1.0)	0/109 (0)	0/112 (0)	0/221 (0)	4	0.9	0.2–2.3
*Taenia* spp.	445	1/76 (1.3)	1/45 (2.2)	2/121 (1.6)	0/69 (0)	0/34 (0)	0/103 (0)	0/109 (0)	0/112 (0)	0/221 (0)	2	0.4	0.1–1.6
Hookworm	445	0/76 (0)	0/45 (0)	0/121 (0)	2/69 (2.9)	0/34 (0)	2/103 (1.9)	6/109 (5.5)	6/112 (5.4)	12/221 (5.4)	14	3.2	1.7–5.2
*Trichostrongylus* spp.-like	445	0/76 (0)	0/45 (0)	0/121 (0)	0/69 (0)	0/34 (0)	0/103 (0)	2/105 (1.9)	3/112 (2.7)	5/221 (2.3)	5	1.1	0.4–2.6
**Total**	**445**	**6/76 (7.9)**	**1/45 (2.2)**	**7/121 (5.8)**	**3/69 (4.4)**	**1/34 (2.9)**	**4/103 (3.9)**	**8/105 (7.6)**	**9/112 (8.0)**	**17/221 (7.7)**	**28**	**6.3**	**4.2**–**9.0**

**Table 2:** Microscopic examination of goat and cattle fecal samples using the Mini Parasep^®^ SF fecal parasite concentrator technique.

Type of ova/cyst	Sample size	Nongkhai			Ratchaburi			Satun			Infected cases	Infection rate (%)	95% CI (%)
		Goat (%)	Cattle (%)	Total (%)	Goat (%)	Cattle (%)	Total (%)	Goat (%)	Cattle (%)	Total (%)			
Strongyle	342	4/7 (71.4)	4/52 (15.4)	8/59 (13.6)	0/4 (0)	7/110 (6.4)	7/114 (6.1)	19/105 (18.1)	3/64 (4.7)	22/169 (13.0)	37	10.8	7.7–14.6
*Trichostrongylus* spp.-like	342	0/7 (0)	0/52 (0)	0/59 (0)	0/4 (0)	0/110 (0)	0/114 (0)	0/105 (0)	0/64 (0)	0/169 (0)	0	0	0.0–1.1
*Trichuris* spp.	342	0/7 (0)	1/52 (2.2)	1/59 (1.7)	0/4 (0)	1/110 (0.9)	1/114 (0.9)	2/105 (1.9)	0/64 (0)	2/169 (1.2)	4	1.2	0.3–3.0
Rumen fluke	342	0/7 (0)	2/52 (0)	2/59 (3.4)	0/4 (0)	4/110 (3.6)	4/114 (3.5)	1/105 (1.0)	6/64 (9.4)	7/169 (4.1)	13	3.8	2.0–6.4
Co-infection of Strongyle and *Trichuris* spp.	342	1/7 (14.3)	2/52 (2.2)	3/59 (5.1)	0/4 (0)	0/110 (0)	0/114 (0)	0/105 (0)	0/64 (0)	0/169 (0)	3	0.9	0.2–2.5
Co-infection of Strongyle and *Trichostrongylus* spp.-like	342	0/7 (0)	1/52 (2.2)	1/59 (1.7)	0/4 (0)	1/110 (0.9)	1/114 (0.9)	21/105 (0)	0/64 (0)	21/169 (12.4)	23	6.7	4.3–9.9
*Eimeria* spp*.*	342	0/7 (0)	2/52 (0)	2/59 (3.4)	0/4 (0)	1/110 (0.9)	1/114 (0.9)	1/105 (1.0)	0/64 (0)	1/169 (0.6)	4	1.2	0.3–3.0
**Total**	**342**	**5/7 (71.4)**	**12/52 (23.1)**	**17/59 (28.8)**	**0/4 (0)**	**14/110 (12.7)**	**14/114 (12.3)**	**44/105 (41.9)**	**9/64 (14.1)**	**53/169 (31.4)**	**84**	**24.6**	**20.1–29.5**

**Table 3:** Microscopic and molecular examination of vegetable sediment samples using the *Trichostrongylus* PCR-RFLP technique.

Parasite eggs/larvae	Nongkhai		Ratchaburi		Satun		Positive samples	Positive (%)	95% CI (%)
	Microscopic (%)	PCR (%)	Microscopic (%)	PCR (%)	Microscopic (%)	PCR (%)			
Hookworm egg	4/23 (17.4)	4/23 (17.4)	0/11 (0)	0/11 (0)	2/40 (12.4)	2/40 (0)	6	8.1	3.0–16.8
*Ascaris* egg	2/23 (8.7)	ND	0/11 (0)	ND	0/40 (0)	ND	2	2.7	0.3–9.4
*Trichuris* egg	0/23 (0)	ND	0/11 (0)	ND	1/40 (2.5)	ND	1	1.4	0.3–7.3
*Taenia* egg	2/23 (8.7)	ND	0/11 (0)	ND	0/40 (0)	ND	2	2.7	0.3–9.4
Unidentified larvae	9/23 (39.1)	ND	3/11 (27.3)	ND	10/40 (25.0)	ND	22	29.7	19.7–41.5
*Trichostrongylus colubriformis*	0/23 (0)	1/23 (4.4)	0/11 (0)	1/11 (9.1)	0/40 (0)	9/40 (22.5)	11	14.9	7.7–25.0
*Trichostrongylus axei*	0/23 (0)	0/23 (0)	0/11 (0)	1/11 (9.1)	0/40 (0)	0/40 (0)	1	1.4	0.3–7.3
*T. colubriformis* and *T. axei*	0/23 (0)	0/23 (0	0/11 (0)	1/11 (9.1)	0/40 (0)	2/40 (5.0)	3	4.1	0.8–11.4

ND = not determined

### Molecular examination and sequencing

**Figure 2:**
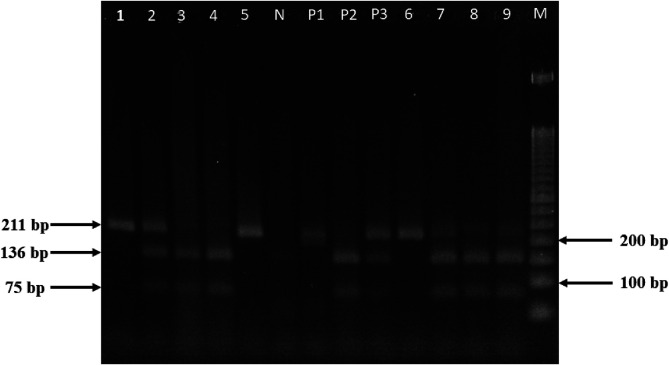
PCR-RFLP analysis of the ITS2 gene. PCR products were visualized on a 3.0% agarose gel. Lane M: 50 bp DNA ladder; Lanes P1–P3: PCR products of *Trichostrongylus axei*, a mixed infection of *T. axei* and *T. colubriformis*, and *T. colubriformis*, respectively, following digestion with *Hinf*I. Lanes 1–9: PCR products from human or livestock stool samples digested with *Hinf*I. Lane N: negative control (nuclease-free water).

PCR analysis of human stool samples revealed *Necator americanus* infection in 3.2% (14/445; 95% CI: 1.7%–5.2%) of participants: 2 cases from Ratchaburi and 12 from Satun. *Trichostrongylus* spp. infection, identified by PCR-RFLP analysis, was detected in 3.4% (15/445; 95% CI: 1.9%–5.5%) of participants, all residing in Satun Province. DNA electrophoresis showed distinct patterns for a selection of infected samples taken from both human and livestock subjects, as visually presented in Figure 2. In Satun, *T. colubriformis* was detected in 12 out of 221 individuals (5.4%; 95% CI: 2.8%–9.3%), and co-infections with *T. colubriformis* and *T. axei* were found in 3 out of 221 individuals (1.4%; 95% CI: 0.3%–3.9%). No cases of *T. axei* monoinfection were observed. Thus, the overall infection rate of human *Trichostrongylosis* in Satun was 6.8% (15/221; 95% CI: 3.8%–11.0%). The molecular findings by location are summarized in Table 4 and Supplementary Table 2.

**Table 4:** Molecular detection of *Trichostrongylus* spp. in human stool samples using the PCR-RFLP technique.

*Trichostrongylus* species	Sample size	Nongkhai			Ratchaburi			Satun			Infected cases	Infection rate (%)	95% CI (%)
		Male (%)	Female (%)	Total (%)	Male (%)	Female (%)	Total (%)	Male (%)	Female (%)	Total (%)			
*Trichostrongylus colubriformis*	445	0/76 (0)	0/45 (0)	0/121 (0)	0/69 (0)	0/34 (0)	0/103 (0)	6/109 (5.5)	6/112 (5.4)	12/221 (5.4)	12	2.7	1.4–4.7
*Trichostrongylus axei*	445	0/76 (0)	0/45 (0)	0/121 (0)	0/69 (0)	0/34 (0)	0/103 (0)	0/109 (0)	0/112 (0)	0/221 (0)	0	0	0.0–0.8
Co-infection of *T. colubriformis* and *T. axei*	445	0/76 (0)	0/45 (0)	0/121 (0)	0/69 (0)	0/34 (0)	0/103 (0)	2/109 (1.8)	1/112 (0.9)	3/221 (1.4)	3	0.7	0.1–2.0
**Total**	**445**	**0/76 (0)**	**0/45 (0)**	**0/121 (0)**	**0/69 (0)**	**0/34 (0)**	**0/103 (0)**	**8/109 (7.3)**	**7/112 (6.2)**	**15/221 (6.8)**	**15**	**3.4**	**1.9–5.5**

In livestock (goats and cattle), *T. colubriformis* and *T. axei* were identified either as single infections or co-infections. The prevalence of *Trichostrongylus* infections in livestock was 13.6% (8/59; 95% CI: 6.0%–25.0%) in Nongkhai, 5.3% (6/114; 95% CI: 2.0%–11.1%) in Ratchaburi, and 20.7%; (35/169; 95% CI: 14.9%–27.6%) in Satun. The molecular results by location and livestock species are presented in Table 5.

**Table 5:** Molecular detection of *Trichostrongylus* spp. in livestock fecal samples using the PCR-RFLP technique.

*Trichostrongylus* species	Sample size	Nongkhai			Ratchaburi			Satun			Infected cases	Infection rate (%)	95% CI (%)
		Goat (%)	Cattle (%)	Total (%)	Goat (%)	Cattle (%)	Total (%)	Goat (%)	Cattle (%)	Total (%)			
*Trichostrongylus colubriformis*	342	4/7 (57.1)	3/52 (5.8)	7/59 (11.9)	1/4 (25)	5/110 (4.5)	6/114 (5.3)	12/105 (11.4)	1/64 (1.6)	13/169 (7.7)	26	7.6	5.0–10.9
*Trichostrongylus axei*	342	0/7 (0)	0/52 (0)	0/59 (0)	0/4 (0)	0/110 (0)	0/114 (0)	6/105 (5.7)	0/64 (0)	6/169 (3.6)	6	1.8	0.6–3.8
Co-infection of *T. colubriformis* and *T. axei*	342	1/7 (14.3)	0/52 (0)	1/59 (1.7)	0/4 (0)	0/110 (0)	0/114 (0)	13/105 (12.4)	3/64 (4.7)	16/169 (9.5)	17	5.0	2.9–7.8
**Total**		**5/7 (71.4)**	**3/52 (5.8)**	**8/59 (13.6)**	**1/4 (25.0)**	**5/110 (4.5)**	**6/114 (5.3)**	**31/105 (29.5)**	**4/64 (6.2)**	**35/169 (20.7)**	**49**	**14.3**	**10.8–18.5**

Molecular identification of vegetable sediment samples showed no detection of hookworm eggs. However, samples from Satun Province tested positive for *Trichostrongylus* eggs. Specifically, *T. colubriformis* was detected in 11 out of 74 samples (14.9%; 95% CI: 7.7%–25.0%), *T. axei* in 1 out of 74 samples (1.4%; 95% CI: 0.3%–7.3%), and co-infections with *T. colubriformis and T. axei* in 3 out of 74 samples (4.1%; 95% CI: 0.8%–11.4%). The molecular results by location are presented in Table 3.

The ITS2 sequences of *T. colubriformis* isolates obtained from humans, livestock, and vegetable sediment samples in this study showed 100% sequence identity within the group. These sequences were submitted to GenBank under accession numbers PQ869638–PQ869642. They also showed 100% similarity with *T. colubriformis* sequences available in the GenBank database, including those from adult worms in human feces from Iran (KY355042) and Lao PDR (KC337069), sheep feces in Australia (MH481561), and adult worms from goats in Lao PDR (AB908959). Similarly, the ITS2 sequences of *T. axei* isolates from livestock and vegetable sediment samples demonstrated 100% identity within the group. These sequences were deposited in GenBank under accession numbers PQ869636–PQ869637. They shared 99% similarity with *T. axei* sequences in GenBank, including those from adult worms in human feces from Thailand (KC337066), goat feces in Australia (MH481571), and larvae from sheep in New Zealand (KC998724).

The ITS1–5.8S rRNA–ITS2 sequences obtained from *N. americanus* isolates derived from human and vegetable sediment samples in this study exhibited 100% similarity within the group. These sequences were deposited in GenBank under accession numbers PQ871212–PQ871214. They also showed 100% identity with *N. americanus* sequences available in the GenBank database, including those from adult worms in human feces from Cambodian migrant workers (OP002314), Laotian individuals (LC036565), and filariform larvae from human feces in Japan (LC036563), as well as a case of human hookworm infection in China (KM891738).

### Benzimidazole resistance gene detection

**Supplementary Figure 1:**
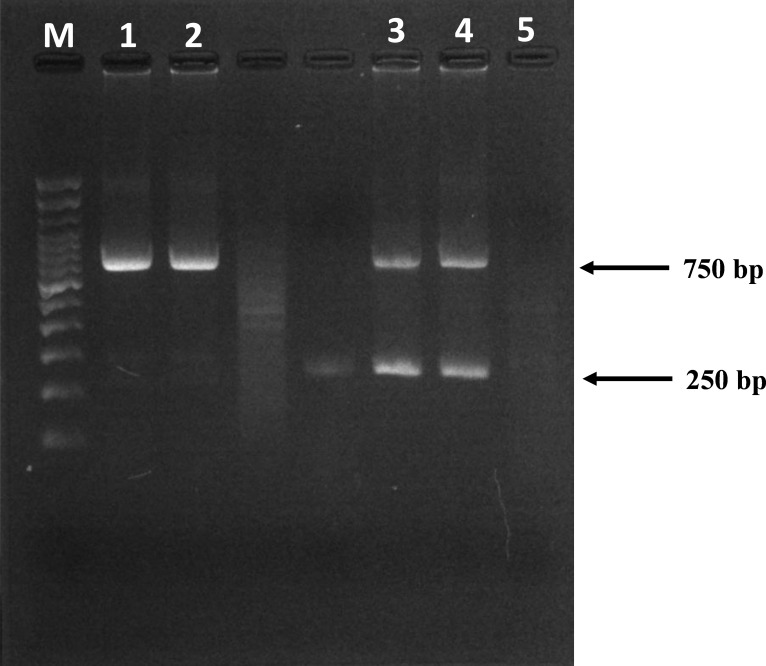
Allele-specific PCR (AS-PCR) amplification of the F200Y single nucleotide polymorphism (SNP) in the isotype 1 β-tubulin gene. PCR products were visualized on a 2.0% agarose gel. Lane M: 100 bp DNA ladder; Lanes 1 and 2: nested PCR products from human samples 4 and 15, respectively; Lanes 3 and 4: AS-PCR products from the same human samples (4 and 15). Lane 5: negative control (AS-PCR), (nuclease-free water).

Allele-specific PCR analysis revealed that *Trichostrongylus* DNA, extracted from successful larval cultures of two human samples (numbers 4 and 15), exhibited benzimidazole-resistant genotypes. Both samples were identified as homozygous resistant (RR) (as shown in Supplementary Figure 1).

### Risk factors

The analysis of risk factors linked to infections of liver flukes, *Taenia* spp., and hookworms in Nongkhai province, as well as hookworm cases in Ratchaburi province and *Trichostrongylus* spp. in Satun province, is detailed in Supplementary Table 1. Univariate analysis demonstrated significant associations between infection and three variables: level of education (*p* = 0.033), occupation (*p* = 0.014), and the use of outdoor hygienic toilets (*p* = 0.018).

The analysis of sociodemographic factors across the three study sites revealed distinct risk profiles, with Satun province – representing Southern Thailand – demonstrating significant correlations between infection status and specific educational and occupational variables. While sex and religion did not significantly influence infection rates in any region (*p* > 0.05), educational attainment was a critical factor in Satun. Specifically, individuals with no formal education in Satun exhibited a significantly higher prevalence of helminth infection (23.1%) compared to those who had completed at least primary school (6.8%; *p* = 0.033). Furthermore, occupation played a pivotal role in the Southern cohort; surprisingly, non-agricultural workers in Satun showed a higher infection rate (15.1%) than those in the agricultural sector (4.9%; *p* = 0.014), a trend not observed in Nongkhai or Ratchaburi.

Sanitation practices and hygiene behaviors further delineated the risk landscape in the southern region. The use of outdoor hygienic toilets was significantly associated with infection status in both Nongkhai (*p* = 0.044) and Satun (*p* = 0.018). In Satun, 10.1% of individuals who never used outdoor hygienic toilets tested positive, whereas no infections were recorded among those who utilized them regularly. Other hygiene factors, including the consumption of raw vegetables, untreated water, and the use of protective footwear (slippers, leather shoes, or boots), did not reach statistical significance in Satun (*p* > 0.05), suggesting that broader environmental or community-level factors may be driving transmission in this locality. Similarly, hand hygiene and food preparation habits showed no significant association with positivity rates across the southern cohort.

## Discussion

The microscopic findings using the Kato–Katz technique in this study were consistent with those of a nationwide survey conducted in Thailand in 2019 [57]. The observed prevalence of major food-borne helminths – *Opisthorchis viverrini*, minute intestinal flukes, and *Taenia* spp. in northeastern Thailand, and the high rate of hookworm infections in the southern and central regions, align with our findings from Nongkhai (northeast), Satun (south), and Ratchaburi (central) Provinces. Variations in helminthic infection patterns are often linked to regional dietary habits and lifestyle behaviors. For instance, in northeastern Thailand, the consumption of raw or undercooked fish dishes (e.g., fermented and pickled fish) [4] and raw minced pork or beef salads [31] increases infection risk. The low prevalence of liver fluke and tapeworm infections among certain groups that consume raw fish and meat may be attributed to a decline in parasite density within their respective hosts. Specifically, research suggests a reduction of metacercariae in fish populations [43], alongside a decrease in intermediate hosts carrying cysticerci. This trend likely stems from modern agricultural shifts, particularly the transition from using manure to chemical fertilizers, which disrupts the traditional parasite life cycle [32]. In southern Thailand, hookworm infections are associated with environmental contamination by infective larvae and behaviors such as walking barefoot on contaminated soil [13, 44]. These findings support the need for a One Health approach to infection control, as recommended by recent studies [33, 36].

The exclusive detection of *Trichostrongylus* species in Satun, compared to its absence in Nongkhai and Ratchaburi, suggests a unique ecological and behavioral interface in Southern Thailand that facilitates zoonotic transmission. Epidemiologically, the southern peninsula is characterized by high annual precipitation and consistent humidity, environmental conditions that are critical for the survival and development of *Trichostrongylus* L3 larvae in the soil. While the northeast and central regions experience more seasonal and arid conditions, the consistently humid southern monsoon climate facilitates year-round environmental contamination [51]. This is consistent with studies in other tropical regions where high humidity and warm temperatures significantly accelerate the development of *Trichostrongyle* eggs into infective larvae [23].

Furthermore, the socio-agricultural landscape of Satun differs significantly from the other study sites. Small ruminant husbandry, particularly goat farming, is more prevalent in Southern Thai communities due to cultural and religious practices. Most goats in Satun are raised in smallholder farms and maintained outdoors under traditional systems, increasing the likelihood of environmental parasitic shedding [28]. Our findings indicate that the transmission risk is not merely occupational but environmental, as evidenced by the significant correlation between infection and the lack of outdoor hygienic toilets in Satun (*p* = 0.018). The presence of these parasites in non-agricultural workers (15.09%) further supports the hypothesis that peri-domestic soil contamination – driven by free-roaming livestock and suboptimal waste management – creates a “spillover” effect into the broader community. This suggests that in Satun, *Trichostrongylus* has established a stable environmental niche that is currently absent in the drier or more industrially farmed regions of Ratchaburi and Nongkhai [45].

A nationwide survey on human helminthiases in Thailand by Wattanawong *et al.* [57] reported three cases of *Trichostrongylus orientalis* infection via microscopic examination, originating from the southern and central regions. In contrast, the present study employing PCR-RFLP analysis, identified only *T. colubriformis* and *T. axei* infections in humans, livestock, and vegetable sediments. In our investigation, we sequenced 10% of positive samples, which revealed the presence of only *T. axei* and *T. colubriformis,* and subsequently utilized the PCR-RFLP to differentiate between *Trichostrongylus* species. While ITS2 sequences for three other *Trichostrongylus* species – *T. vitrinus*, *T. retortaeformis*, and *T. tenuis* – are available in the GenBank database [25], these sequences, like that of *T. axei*, are not cleaved by the *Hinf*I restriction enzyme. We also noted that the ITS2 sequence for *T. orientalis* was unavailable in the GenBank database. For future research, particularly when investigating co-infections, the use of nemabiome metabarcoding targeting the ITS2 region is recommended for a more comprehensive analysis [14].

Although *T. orientalis* was historically recognized as a major species infecting both humans and livestock in Iran [18], more recent Iranian studies confirmed *T. colubriformis* and *T. axei* as the dominant species in human cases [46–48]. These findings underscore the importance of integrating molecular techniques such as PCR-RFLP in future nationwide surveys in Thailand. Such methods will enhance the accuracy of species-level identification and provide a clearer understanding of the current epidemiological landscape of *Trichostrongylus* infections.

The results of this study present the first reported cases of co-infection with *T. colubriformis* and *T. axei* in humans in Thailand. Although previous studies in northeastern Thailand and Lao PDR have confirmed the presence of both species in human infections using PCR techniques, co-infections involving these two species have not previously been documented [41, 45, 58]. Interestingly, studies in Iran have reported co-infections involving *T. colubriformis* and *T. vitrinus*, as well as *T. vitrinus* and *T. longispicularis* [47].

Our study confirmed the presence of *T. axei* and *T. colubriformis* in both cattle and goats, aligning with findings from Malaysia, Iran, and countries in Africa [1, 55]. Additionally, our detection of the F200Y mutation in the isotype 1 β-tubulin gene of *T. colubriformis* third-stage larvae from human fecal cultures – using allele-specific PCR – supports previous reports of benzimidazole resistance in *Trichostrongylus* species in livestock [12]. Due to the emergence of anthelmintic resistance, ongoing surveillance of benzimidazole efficacy in treating human trichostrongylosis is warranted.

In this study, PCR demonstrated a 3-fold increase in detection yield compared to microscopy across all sample types. This discrepancy highlights the higher sensitivity of molecular techniques; while microscopy remains the “gold standard” for visualizing morphology, it is limited by a high threshold of detection. The 10 additional cases identified by PCR likely represent low-intensity infections or environmental contamination (in the case of vegetables) that fell below the visual threshold of microscopic examination.

One fecal sample that tested positive for *Trichostrongylus* eggs by microscopy (sample No. 17) yielded a negative result in the PCR assay. This discrepancy may be attributed to several factors. First, the presence of PCR inhibitors in the fecal sample – previously reported as a common issue [53] – could have interfered with DNA amplification, resulting in a false-negative outcome. Second, the sample may have contained a low parasitic burden, providing insufficient DNA template for successful amplification.

Our study also revealed a significant association between lower levels of education and increased risk of trichostrongylosis. Individuals with no formal education exhibited markedly higher infection rates (23.1%) compared to those with at least primary school education (6.8%) (*p* = 0.033). This finding aligns with previous research on soil-transmitted helminth infections, which has demonstrated strong correlations between low educational attainment and higher infection risk [44]. However, it is important to recognize that this association may not be apply universally across all parasitic infections. For instance, a study in northeastern Thailand found no significant link between education level and infections with *O. viverrini* or *Strongyloides stercoralis* [10]. This suggests that the influence of education on infection risk may vary depending on the parasite species and the broader socio-economic and environmental context of the population studied.

Individuals with lower levels of education may have limited awareness of proper hygiene practices, reduced access to adequate sanitation, and restricted exposure to accurate health information. These factors can significantly increase their risk of exposure to and infection with soil-transmitted helminths [22].

Based on our findings, a significant association exists between sanitation practices and the prevalence of trichostrongylosis infection. Specifically, individuals who reported never using outdoor hygienic toilets had a dramatically higher infection rate compared to those who used such facilities sometimes or always, a difference that proved statistically significant (*p* = 0.018). This elevated risk is consistent with prior research linking helminth infections to poor sanitary behaviors, such as inadequate hand washing after using the toilet (*p* < 0.250) [58]. The mechanism of infection is likely the contamination of non-hygienic toilet environments with infective *Trichostrongylus* larvae, which are then transmitted to humans through the fecal-oral route, primarily via insufficient hand washing before consuming food. Conversely, a significantly higher prevalence of liver fluke and taeniasis infections (33.3%) was observed among individuals who did use outdoor hygienic toilets compared to those who did not (*p* = 0.044). This apparent contradiction may reflect differences in the biology and transmission pathways of these parasites. *Trichostrongylus* larvae are typically transmitted through contact with contaminated soil or ingestion of contaminated vegetation, whereas liver fluke and taeniasis infections are commonly associated with the consumption of raw or undercooked fish and meat.

Previous studies have reported human trichostrongylosis among farmer populations [42, 52] and sheepherders [15]. Established risk factors include close contact with livestock [58], handling animal feces – particularly when processing goat manure for fertilizer [7] – and the consumption of raw or undercooked vegetables or water contaminated with livestock manure [45]. Notably, our study also identified an association between *Trichostrongylus* infection and non-agricultural occupations, including individuals working in business, government, and the fishing industry. Although the study primarily targeted goat farmers and their families, approximately one-third of the questionnaire respondents were not directly involved in goat farming, although they still owned livestock. This unexpected finding suggests the need for further investigation. In-depth interviews with infected individuals are recommended to better understand their specific exposure routes and risk behaviors [21].

This study has several limitations. First, the inability to recover adult *Trichostrongylus* worms from human stool samples restricted our capacity to perform morphological identification. Since all infections were confirmed using molecular techniques prior to treatment, opportunities for worm recovery were limited. Second, although contact with livestock feces is a recognized risk factor for *Trichostrongylus* infection, this aspect was not explicitly investigated due to constraints in the questionnaire design. Third, the unidentified larvae recovered from vegetable samples were not subjected to larval culture or molecular identification. These larvae may have been free-living nematodes or zoonotic parasites such as *Strongyloides* spp. [9].

## Conclusion

In conclusion, this study offers important insights into the epidemiology and risk factors associated with human trichostrongylosis in Thailand. In Satun, a total of 221 residents were tested for *Trichostrongylus* infection, revealing that 5.4% had a single infection with *T. colubriformis*. A smaller proportion, 1.4% showed a co-infection involving both *T. colubriformis* and *T. axei*. Levels of education, occupation, and the use of outdoor toilets were identified as key risk factors. The findings underscore the need for integrated surveillance and targeted control strategies to mitigate the public health impact of this neglected zoonotic disease.

## Supplementary material

*Supplementary Table 1*: Factors associated with helminth infections in humans from three locations. *Supplementary Table 2*: Profiles of 16 human trichostrongylosis cases in La-ngu Subdistrict, Satun Province. The supplementary material of this article is available at https://www.parasite-journal.org/10.1051/parasite/2026027/olm.
