# Supplementary material for: Prevalence and risk factors of human trichostrongylosis in Satun, southern Thailand
Source: Parasite. 2026 May 25;33:29. doi: 10.1051/parasite/2026027 (PMC13200820; doi:10.1051/parasite/2026027)
Supplement: Supplementary file 1 — Supplementary Table 1: Factors associated with helminth infections in humans from three locations. [file parasite-33-29-s1.pdf]

Supplementary Table 1. Factors associated with helminth infections in humans from three locations.

Items	Nongkhai		*p* value	Ratchaburi		*p* value	Satun		*p* value
	Liver fluke, pork tapeworm and hookworm infection			Hookworm infection			Trichostongylois		
	Positive case	Negative cases		Positive case	Negative cases		Positive case	Negative cases	
Sex			0.196			0.526			0.846
- male	6/76 (7.89%)	70/76 (92.11%)		4/69 (5.80%)	65/69 (94.20%)		8/109 (7.34%)	101/109 (92.66%)	
- female	1/45 (2.22%)	44/45 (97.78%)		1/34 (2.94%)	33/34 (97.06%)		9/112 (8.04%)	103/112 (91.96%)	
Religion			0.939			0.747			0.375
- Buddhism	7/118 (5.93%)	111/118 (94.07%)		5/101 (4.95%)	96/101 (95.05%)		0/9 (0%)	9/9 (100%)	
- Islam	0/1 (0%)	1/1 (100%)		0/2 (0%)	2/2 (100%)		17/211 (8.06%)	194/211 (91.94%)	
- Christianity	0/1 (0%)	1/1 (100%)		-			-	-	
Education			0.614			ND			0.033
- no education	0/4 (0%)	4/4 (100%)		-	-		3/13 (23.08%)	10/13 (76.92%)	
- at least primary	7/117 (5.98%)	110/117 (94.02%)		5/99 (5.05%)	94/99 (94.95%)		14/206 (6.80%)	192/206 (93.20%)	

school									
Occupation			0.335			0.335			0.014
- agricultural	7/104 (6.73%)	97/104 (93.27%)		5/85 (5.88%)	80/85 (94.12%)		8/163 (4.91%)	155/163 (95.09%)	
- non-agricultural	0/13 (0%)	13/13 (100%)		0/15 (0%)	15/15 (100%)		8/53 (15.09%)	45/53 (84.91%)	
Toilet			0.795			ND			0.436
- not using	0/1 (0%)	1/1 (100%)		-	-		0/7 (0%)	7/7 (100%)	
- use	7/111 (6.31%)	104/111 (93.69%)		5/100 (5%)	95/100 (95%)		17/213 (7.98%)	196/213 (92.02%)	
Raw vegetable and untreated water			0.664			0.496			0.375
- consume	6/101 (5.94%)	95/101 (94.06%)		5/91 (5.49%)	86/91 (94.51%)		17/211 (8.06%)	194/211 (91.94%)	
- not consume	0/3 (0%)	3/3 (100%)		0/8 (0%)	8/8 (100%)		0/9 (0%)	9/9 (100%)	
Untreated water			0.496			0.223			0.139
- consume	3/68 (4.41%)	65/68 (95.59%)		5/78 (6.41%)	73/78 (93.59%)		12/187 (6.42%)	175/187 (93.58%)	
- not consume	2/25 (8%)	23/25 (92%)		0/22 (0%)	22/22 (100%)		4/28 (14.29%)	24/28 (85.71%)	

Slipper			0.721			ND			0.682
- not use	7/117 (5.98%)	110/117 (94.02%)		5/103 (4.85%)	98/103 (95.15%)		17/219 (7.76%)	202/219 (92.24%)	
- sometime/always use	0/2 (0%)	2/2 (100%)		-	-		0/2 (0%)	2/2 (100%)	
Leather shoes			0.661			0.819			0.719
- not use	7/116 (6.03%)	109/116 (93.97%)		5/100 (5%)	95/100 (95%)		16/202 (7.92%)	186/202 (92.08%)	
- sometime/always use	0/3 (0%)	3/3 (100%)		0/1 (0%)	1/1 (100%)		1/18 (5.56%)	17/18 (94.44%)	
Boots			ND			0.837			0.406
- not use	7/119 (5.88%)	112/119 (94.12%)		4/98 (4.08%)	94/98 (95.92%)		16/214 (7.48%)	198/214 (92.52%)	
- sometime/always use	-	-		0/1 (0%)	1/1 (100%)		1/6 (16.67%)	5/6 (83.33%)	
Hand washing before cooking and meals			ND			0.820			ND
- never	6/118 (5.08%)	112/118 (94.92%)		5/102 (4.90%)	97/102 (95.10%)		17/219 (7.76%)	202/219 (92.24%)	
- sometime/always use	-	-		0/1 (0%)	1/1 (100%)		-	-	
Cutting board and knife cleansing before cooking			ND			0.820			0.556

- never	6/117 (5.13%)	111/117 (94.87%)		5/102 (4.90%)	97/102 (95.10%)		17/213 (7.98%)	196/213 (92.02%)	
- sometime/always use	-	-		0/1 (0%)	1/1 (100%)		0/4 (0%)	4/4 (100%)	
Washing fruit and vegetable before eating			ND			0.820			ND
- never	7/117 (5.98%)	110/117 (94.02%)		5/102 (4.90%)	97/102 (95.10%)		17/219 (7.76%)	202/219 (92.24%)	
- sometime/always use	-	-		0/1 (0%)	1/1 (100%)		-	-	
Drinking treated water			0.719			0.421			0.091
- never	3/42 (7.14%)	39/42 (92.86%)		1/41 (2.44%)	40/41 (97.56%)		9/74 (12.16%)	65/74 (87.84%)	
- sometime/always use	4/73 (5.48%)	69/73 (94.52%)		3/51 (5.88%)	48/51 (94.12%)		8/142 (5.63%)	134/142 (94.37%)	
Eating uncooked meal			0.693			0.820			0.193
- never	2/38 (5.26%)	36/38 (94.74%)		5/102 (4.90%)	97/102 (95.10%)		16/215 (7.44%)	199/215 (92.56%)	
- sometime/always use	3/81 (3.70%)	78/81 (96.30%)		0/1 (0%)	1/1 (100%)		1/4 (25%)	3/4 (75%)	
Outdoor hygienic toilet			0.044			0.837			0.018
- never use	6/113 (5.31%)	107/113 (94.69%)		4/98 (4.08%)	94/98 (95.92%)		17/168 (10.12%)	151/168 (89.88%)	
- sometime/always use	1/3 (33.33%)	2/3 (66.67%)		0/1 (0%)	1/1 (100%)		0/51 (0%)	51/51 (100%)	

Excretion outside the toilet			0.782			0.469			0.269
- never	2/35 (5.71%)	33/35 (94.29%)		1/49 (2.04%)	48/49 (97.96%)		11/113 (9.73%)	102/113 (90.27%)	
- sometime/always	5/70 (7.14%)	65/70 (92.86%)		2/42 (4.76%)	40/42 (95.24%)		6/105 (5.71%)	99/105 (94.29%)	
Washing hands after toilet usage			ND			ND			0.402
- never	7/116 (6.03%)	109/116 (93.97%)		5/101 (4.95%)	96/101 (95.05%)		17/210 (8.10%)	193/210 (91.90%)	
- sometime/always use	-	-		-			0/8 (0%)	8/8 (100%)	
History of stool examination			0.643			0.476			0.849
- uncertain/never	5/74 (6.76%)	69/74 (93.24%)		5/93 (5.38%)	88/93 (94.62%)		15/198 (7.58%)	183/198 (92.42%)	
- ever	2/43 (4.65%)	41/43 (95.35%)		0/9 (0%)	9/9 (100%)		2/23 (8.70%)	21/23 (91.30%)	
History of anthelmintic drug used			0.289			0.360			0.377
- uncertain/never	6/79 (7.59%)	73/79 (92.41%)		5/88 (5.68%)	83/88 (94.32%)		17/212 (8.02%)	195/212 (91.98%)	
- ever	1/38 (2.63%)	37/38 (97.37%)		0/14 (0%)	14/14 (100%)		0/9 (0%)	9/9 (100%)	
History of protozoa drug used			0.745			0.425			0.436

-	uncertain/never	6/94 (6.38%)	88/94 (93.62%)	5/91 (5.49%)	86/91 (94.51%)	17/213 (7.98%)	196/213 (92.02%)
-	ever	1/22 (4.54%)	21/22 (95.46%)	0/11 (0%)	11/11 (100%)	0/7 (0%)	7/7 (100%)

ND = not determined
