# Supplementary material for: Prevalence and risk factors of human trichostrongylosis in Satun, southern Thailand
Source: Parasite. 2026 May 25;33:29. doi: 10.1051/parasite/2026027 (PMC13200820; doi:10.1051/parasite/2026027)
Supplement: Supplementary file 2 — Supplementary Table 2: Profiles of 16 human trichostrongylosis cases in La-ngu Subdistrict, Satun Province. [file parasite-33-29-s2.pdf]

Supplementary Table 2. Profiles of 16 human trichostrongylosis cases in La-ngu Subdistrict, Satun Province.

No.	Sex	Age (year	Village name	Kato–Katz examination result	Human PCR fecal examination result	Livestock Mini Parasep examination result	Livestock PCR fecal examination result
1	M	83	Tha Chamuang	negative	*T. colubriformis*	strongyle	*T. colubriformis*
2	F	43	Tha Chamuang	negative	*T. colubriformis*	negative	negative
3	M	51	Tha Chamuang	negative	*T. colubriformis* and *T. axei*	negative	negative
4*	M	67	Bakan Tohtid	positive	*T. colubriformis*	negative	*T. colubriformis* and *T. axei*
5	F	59	Lom Puen	negative	*T. colubriformis*	negative	negative

					and *T. axei*		
6	M	ND	Khlong Kut	positive	*T. colubriformis*	negative	*T. colubriformis*
7	M	62	Khlong Kut	negative	*T. colubriformis*	negative	*T. colubriformis* and *T. axei*
8	F	49	La-ngu	negative	*T. colubriformis*	negative	*T. colubriformis*
9	M	44	Hui Mapraw	negative	*T. colubriformis*	strongyle	*T. colubriformis*
10	M	ND	Hui Mapraw	negative	*T. colubriformis*	rumen fluke	*T. colubriformis* and *T. axei*
11	F	63	Baan Tung Pattana	negative	*T. colubriformis*	negative	*T. colubriformis* and *T. axei*
12	M	61	Hua Tang	negative	*T. colubriformis* and *T. axei*	negative	*T. colubriformis* and *T. axei*
13	F	46	Khlong Nam Khem	negative	*T. colubriformis*	negative	negative

14	F	35	Baan Tung Pattana	positive	*T. colubriformis*	negative	negative
15*	F	57	Baan Tung Pattana	positive	*T. colubriformis*	negative	negative
16	F	24	Baan Nai Mueang	positive	negative	negative	negative

* Fecal samples were subjected to culture, and F200Y polymorphism in the isotype 1 β-tubulin gene was investigated.

ND = no data
